# 2537: Usability and adoption of the first enterprise-wide app prescribing platform, RxUniverse, in an academic tertiary care hospital

**DOI:** 10.1017/cts.2017.152

**Published:** 2018-05-10

**Authors:** Sonya Makhni, Daniel Tuchman, Farah Fasihuddin, Jason Rogers, Ashish Atreja

**Affiliations:** Icahn School of Medicine at Mount Sinai, New York, NY, USA

## Abstract

OBJECTIVES/SPECIFIC AIMS: To assess the usability and adoption of RxUniverse, a novel platform that enables health care providers to directly disseminate proven, evidence-based mobile health apps to patients. METHODS/STUDY POPULATION: Among 5 pilot clinical sites, 40 physicians and front-line providers consisting of medical assistants and receptionists were trained on the RxUniverse platform. They were instructed on the platform’s purpose, were shown a demonstration of the functionality, and were observed in a trial process of prescribing an app. Specific implementation plans were designed with the help of the clinic staff in order to best fit in with their present workflows. The well-validated System Usability Score (SUS) was used to assess the usability of the platform. Prescriptions of 100 relevant app prescriptions within a 8-week pilot period was set as the adoption goal. RESULTS/ANTICIPATED RESULTS: Within the pilot period, greater than 2000 apps were prescribed across all users. Of the 40 providers trained on the RxUniverse platform, 26 prescribed >5 apps during the trial period. Of these 26 individuals, 18 prescribed >20 apps, 14 prescribed >50 apps, and 5 prescribed >80 apps; 58% of users reported frequent use (weekly or daily) of the platform. In total, 19 responses were received for the SUS survey. The RxUniverse platform received a usability score of 82%. DISCUSSION/SIGNIFICANCE OF IMPACT: As the pace of innovation continues to accelerate, health care providers will need to quickly integrate new digital-based tools into their workflows, and patients will need to be able to easily and readily access these tools. RxUniverse provides the necessary mechanisms, user-friendly interface, and EHR integration functionality to accomplish this. The total number of apps prescribed surpassed 2000, which far exceeded the initial target of 100 apps. The platform also scored an 82% on the SUS, which is considered an “A” by industry standards. By comparison, other health apps considered to have to be in the highest-rating groups have reported scores of 77.5% and an overall average of 68% among all systems. These outcomes demonstrate the high adoption and usability of the RxUniverse platform, an important platform that can be used to prescribe the latest technologies directly to patients.

